# Supplementary figures and images for: Associative Overdominance and Negative Epistasis Shape Genome-Wide Ancestry Landscape in Supplemented Fish Populations
Source: Genes (Basel). 2021 Apr 3;12(4):524. doi: 10.3390/genes12040524 (PMC8065892; doi:10.3390/genes12040524)

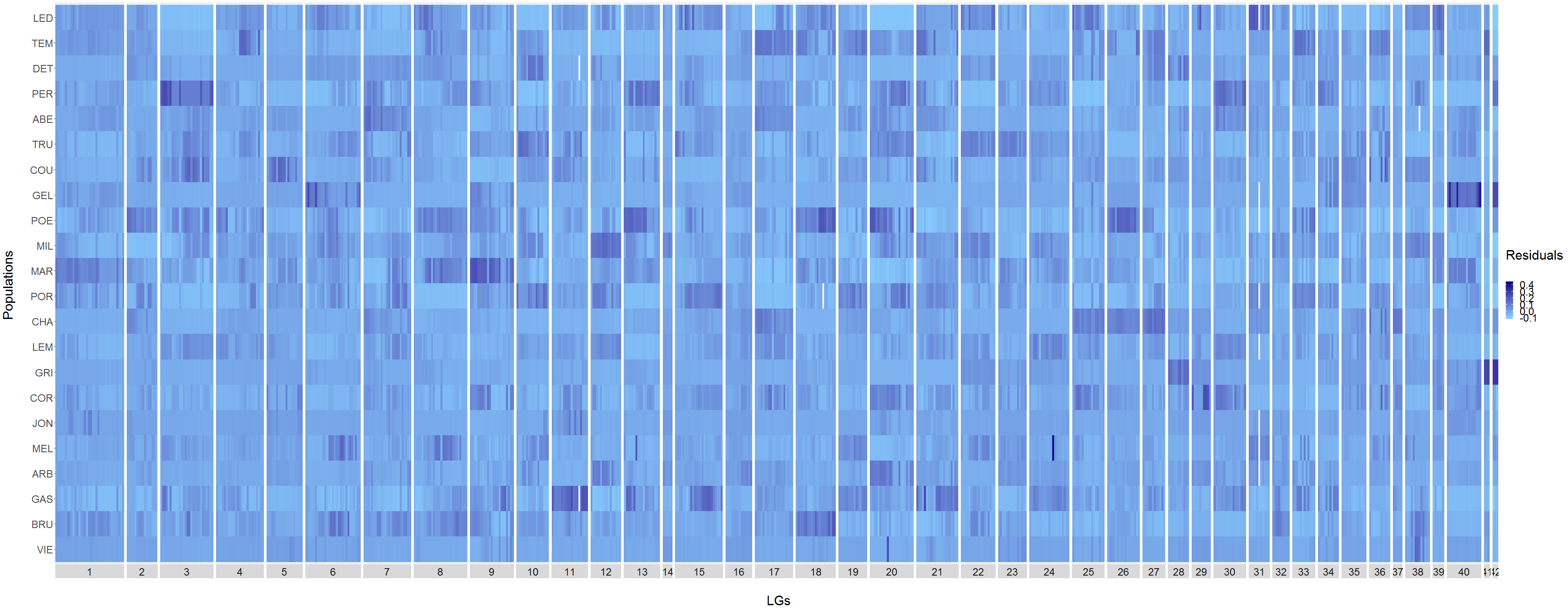

Supplement: Supplementary file 1 [file genes-12-00524-s001.zip › Figure_Sup2.png]

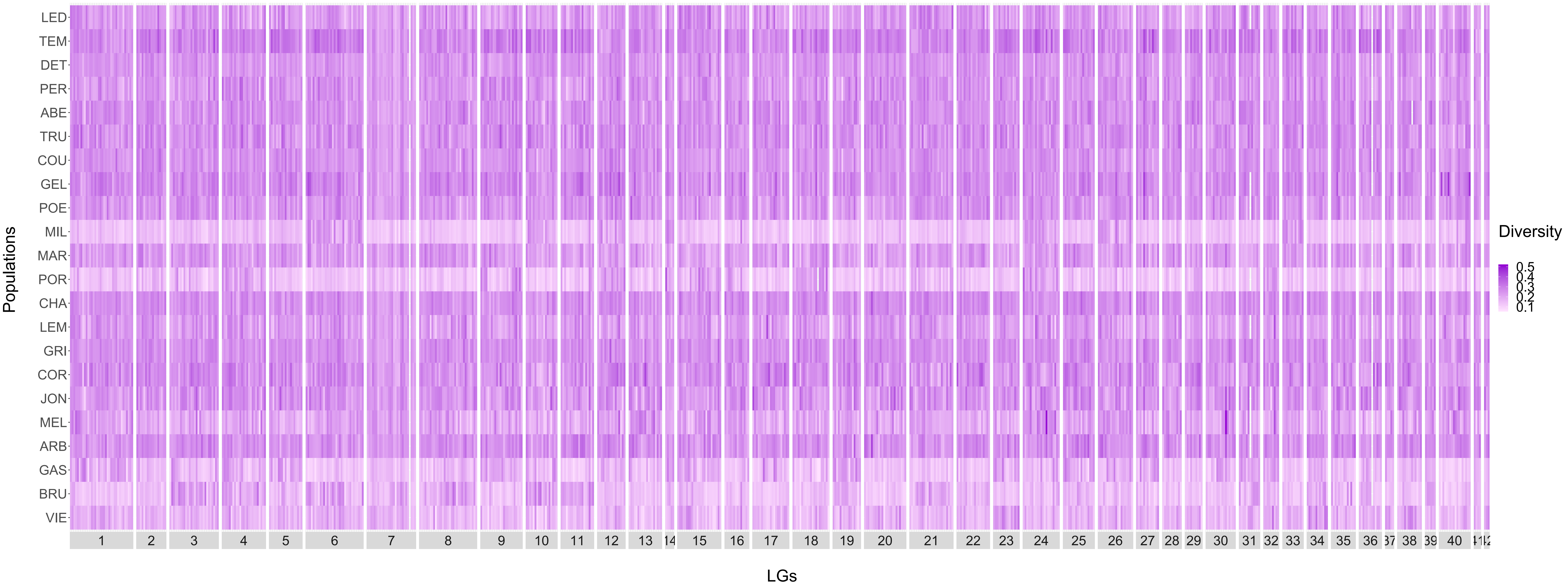

Supplement: Supplementary file 1 [file genes-12-00524-s001.zip › Figure_Sup3.pdf]

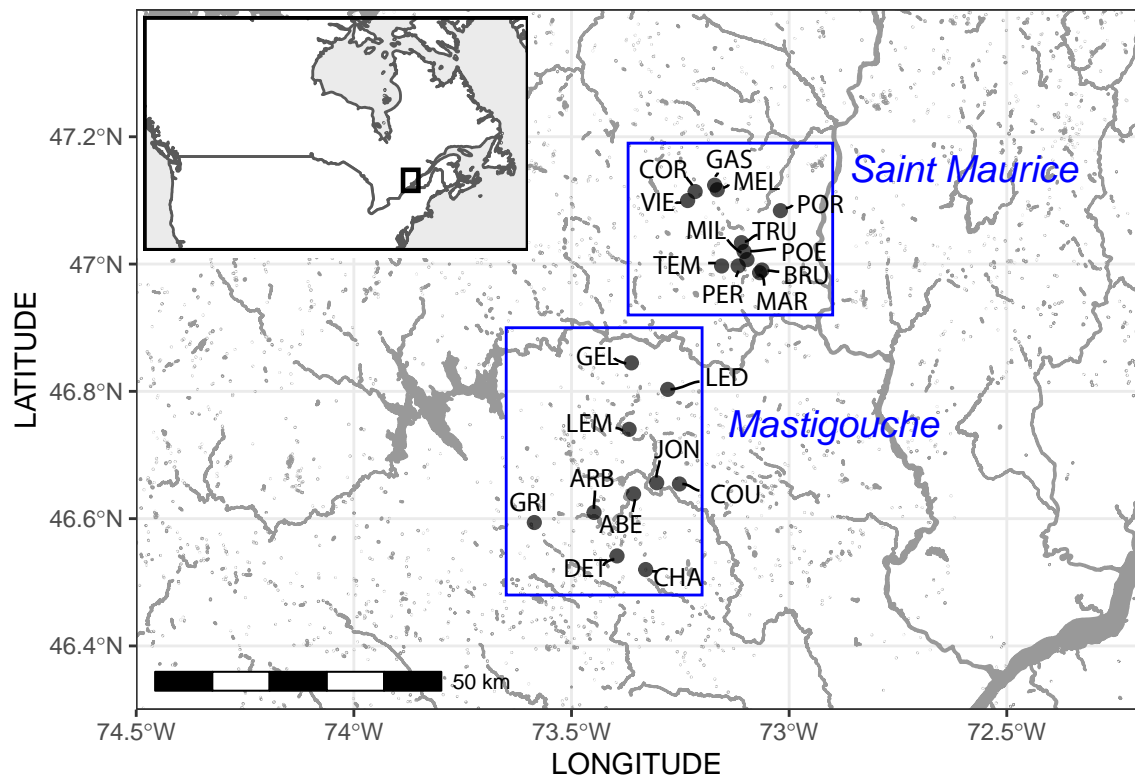

Supplement: Supplementary file 1 [file genes-12-00524-s001.zip › Figure_Sup1.pdf]
